# Exploring the protective role of social support on PTSD, depression, and anxiety among internally displaced persons residing in Shelters Amidst War in Sudan: A cross-sectional study

**DOI:** 10.1097/MD.0000000000048343

**Published:** 2026-04-10

**Authors:** Mohammed Salah Alfahal

**Affiliations:** a Research and Development Center, Psychiatry Council, Sudan Medical Specialization Board, Khartoum, Sudan.

**Keywords:** anxiety, depression, internally displaced persons, PTSD, social support, Sudan

## Abstract

Armed conflict and forced displacement in Sudan have created major disruptions to social and health systems. Social support may be differentially associated with mental health among internally displaced persons (IDPs), yet context-specific evidence remains limited. This study examined the association between perceived social support and symptoms of post-traumatic stress, depression, and anxiety among IDPs in collective shelters in Port Sudan. An analytical cross-sectional study was conducted among 207 adult IDPs recruited through convenience sampling across multiple collective shelters. Structured face-to-face interviews were conducted in Arabic. Perceived social support was assessed using the Oslo Social Support Scale (OSSS-3). Post-traumatic stress symptoms were measured using the Primary Care PTSD Screen for DSM-5 (PC-PTSD-5), depressive symptoms using the Patient Health Questionnaire-9 (PHQ-9), and anxiety symptoms using the generalized anxiety disorder-7 (GAD-7). Associations were examined using unadjusted 2-tailed bivariate correlation analyses, with statistical significance defined as *P* < .05. Participants were predominantly female (90.4%) with marked socioeconomic vulnerability (84.1% unemployed). Mean (±SD) scores were OSSS-3: 7.69 ± 2.51, PC-PTSD-5: 1.98 ± 1.40, PHQ-9: 9.86 ± 6.08, and GAD-7: 8.46 ± 5.53. Higher perceived social support was moderately associated with fewer post-traumatic stress symptoms (*r* = −0.328, *P* < .01). Participants screening positive for probable post-traumatic stress disorder (PTSD) (PC-PTSD-5 ≥ 3) were more likely to report poor social support. Associations between social support and depression and anxiety were weak and non-significant. Higher perceived social support was associated with fewer post-traumatic stress symptoms, while links with depression, anxiety, and service use were non-significant. Findings highlight heterogeneity in displacement-related distress and support community-based, resilience-oriented mental health approaches alongside efforts to reduce systemic barriers to care.

## 1. Introduction

Armed conflict and forced displacement constitute major global public health challenges with far-reaching consequences for affected populations. In Sudan, the humanitarian situation deteriorated sharply following the escalation of armed conflict in April 2023, resulting in widespread insecurity, disruption of social structures, and severe strain on essential services. By early 2024, the crisis had generated large-scale forced displacement, with more than 8 million people displaced within Sudan and across neighboring countries, placing substantial pressure on humanitarian, social, and health systems.^[1,2]^ Within Sudan, displacement has been directed predominantly toward relatively safer eastern and northern states, including Red Sea State, Kassala, and Gedaref. Port Sudan, the capital of Red Sea State and Sudan’s principal seaport, has emerged as a major destination for internally displaced persons (IDPs) due to its relative security, functional transport infrastructure, and role as an administrative and humanitarian coordination hub. Large numbers of displaced individuals have settled in the city and its surrounding areas, residing with host communities, in rented housing, or in temporary collective shelters such as schools and public buildings.^[3]^ The rapid influx of IDPs has placed significant pressure on local infrastructure, housing, and health services, often resulting in overcrowded living conditions and limited access to basic services. These circumstances create environments of sustained adversity that increase vulnerability to psychological distress among conflict-affected individuals.

Exposure to armed conflict and displacement is associated with a range of adverse mental health outcomes, although individual responses vary widely. Experiences such as violence, loss of livelihoods, disruption of social networks, and prolonged uncertainty may contribute to symptoms of post-traumatic stress, depression, and anxiety.^[4,5]^ Importantly, exposure to traumatic events and post-traumatic stress disorder (PTSD) are not synonymous. PTSD represents a potential outcome of trauma exposure rather than an inevitable consequence, and many conflict-affected individuals demonstrate resilience and adaptive coping despite significant adversity.^[6]^ Studies among displaced populations report wide variability in the prevalence of post-traumatic stress symptoms and other common mental health conditions, reflecting contextual, cultural, and methodological differences.^[7,8]^

In Sudan, the psychological impact of conflict is further compounded by profound barriers to accessing mental health care. Since April 2023, large proportions of health facilities have become nonfunctional or only partially functional due to insecurity, damage, and shortages of essential supplies and personnel.^[9]^ Mental health services, which were already limited and largely centralized in Khartoum prior to the conflict, have been particularly affected, resulting in severe disruptions to specialist care, referral pathways, and continuity of treatment. Attacks on health facilities, displacement of healthcare workers, and restricted humanitarian access have further undermined service delivery.^[10]^ In eastern Sudan, including Red Sea State, the rapid influx of IDPs has placed additional strain on already overstretched health services, while mental health and psychosocial support services have not expanded proportionally to meet increased demand.^[1]^ Consequently, many individuals experiencing psychological distress remain without access to timely and appropriate support.

Within this context, social support has been identified as a key factor influencing psychological wellbeing in situations of adversity. The stress-buffering hypothesis proposes that supportive social relationships can mitigate the negative effects of stressful experiences by providing emotional, informational, and practical resources.^[11]^ From an ecological systems perspective, mental health is shaped by interactions across multiple levels, including family, community, and broader sociocultural environments. Empirical evidence from refugee and displacement settings suggests that stronger perceived social support is associated with fewer post-traumatic stress symptoms and improved coping, whereas weak or disrupted support networks are linked to poorer mental health outcomes.^[12,13]^ However, displacement and ongoing conflict in Sudan have fragmented many traditional social structures, potentially limiting the protective role of social support.^[3]^

Despite growing international evidence on mental health and psychosocial wellbeing among displaced populations, context-specific research from Sudan remains limited.^[1]^ Few studies have examined the association between perceived social support and mental health outcomes among Sudanese IDPs using validated instruments such as the Oslo Social Support Scale (OSSS-3), the Primary Care PTSD Screen for DSM-5 (PC-PTSD-5), the Patient Health Questionnaire-9 (PHQ-9), and the generalized anxiety disorder-7 (GAD-7) (14–15). Moreover, there is limited quantitative evidence exploring how social support interacts with demographic and socioeconomic factors to shape psychological distress in low-resource, conflict-affected settings.

This study aims to address these gaps by examining the association between perceived social support and symptoms of post-traumatic stress, depression, and anxiety among IDPs residing in shelters in Port Sudan. By applying validated assessment tools within a theoretically informed framework, the study seeks to generate evidence that can inform culturally sensitive, community-based mental health and psychosocial support (MHPSS) interventions and guide humanitarian programming and policy development for conflict-affected populations in Sudan and comparable settings.

## 2. Methods

### 2.1. Study design

This study employed an analytical cross-sectional design to examine the association between perceived social support and mental health symptom severity – specifically post-traumatic stress symptoms, depressive symptoms, and anxiety symptoms – among IDPs residing in collective shelters in Port Sudan, Sudan. A cross-sectional approach was selected due to the ongoing armed conflict, population mobility, and operational constraints that limited the feasibility of longitudinal follow-up. Such designs are widely used in humanitarian and conflict settings to generate timely, context-specific evidence. However, this design does not allow causal inference, and all findings are interpreted as associative rather than causal.

### 2.2. Study setting

The study was conducted in Port Sudan, the capital of Red Sea State in eastern Sudan. Following the escalation of armed conflict in April 2023, Port Sudan became a major destination for internally displaced populations due to its relative security, functioning seaport, and concentration of governmental and humanitarian services. Large numbers of displaced individuals sought refuge in urban collective shelters, placing substantial strain on local infrastructure and basic services.

Data collection took place in multiple collective shelters distributed across different areas of Port Sudan. These shelters consisted primarily of public buildings repurposed for displacement, including schools (e.g., Othman Digna schools), student dormitories (including female dormitories), youth hostels, Civil Defense facilities, port-adjacent buildings, and large collective shelter compounds with multiple sections (e.g., Al-Qashiya A–D). Shelters were located across eastern and western residential sectors, industrial zones, and port-adjacent areas. Living conditions were characterized by overcrowding, limited privacy, shared sanitation facilities, constrained access to basic services, and for many residents, prolonged displacement, all of which are recognized contributors to psychological distress among conflict-affected populations.

### 2.3. Participants and eligibility criteria

Participants were internally displaced adults aged 18 years and older who were residing in the selected shelters at the time of data collection. Eligibility criteria included the ability to communicate in Arabic and sufficient cognitive capacity to understand the study information and provide informed consent. Individuals who were acutely medically unwell or unable to participate due to significant cognitive impairment were not included. Participation was voluntary, and no incentives were provided.

### 2.4. Sampling technique and sample size

A convenience sampling strategy was used due to security constraints, population mobility, and limited access windows in the humanitarian context. Although this non-probability sampling approach limits generalizability, it enabled access to a vulnerable population during an acute crisis.

To enhance diversity within the accessible population, recruitment was conducted across multiple shelters, at different times of the day, and across different shelter zones when feasible. Both men and women were approached; however, participation was predominantly female.

A total of 207 participants completed the interviews. The resulting sample was largely female (90.4%), unemployed (84.1%), and of low income (78.4%). These characteristics reflect individuals accessible within collective shelters during the study period rather than the entire displaced population in Port Sudan. The study does not estimate the proportion of the total displaced population represented by the sample, and findings should be interpreted accordingly.

### 2.5. Data collection procedures

Data was collected through structured face-to-face interviews conducted in Arabic, the participants’ primary language. Interviews were conducted by trained data collectors with experience working in humanitarian and displacement settings. To ensure privacy and confidentiality, interviews were conducted in quiet areas within shelters where conversations could not be overheard. Data collectors followed standardized protocols and received training on ethical conduct, informed consent, and psychosocial sensitivity when engaging with conflict-affected individuals.

## 3. Measures

### 3.1. Perceived social support

Perceived social support was assessed using the Oslo Social Support Scale (OSSS-3), a three-item measure widely used in population-based and humanitarian research.^[14]^ Total scores range from 3 to 14 and were categorized as poor (3–8), moderate (9–11), or strong (12–14) social support.

### 3.2. Post-traumatic stress symptoms

Post-traumatic stress symptoms were measured using the Primary Care PTSD Screen for DSM-5 (PC-PTSD-5), a validated screening instrument designed to identify probable PTSD symptoms in primary care and humanitarian contexts. A cutoff score of ≥3 was used to indicate a positive screen for probable PTSD, consistent with recommended thresholds in humanitarian screening contexts.^[15]^ The PC-PTSD-5 assesses symptom presence and does not constitute a clinical diagnosis. Trauma exposure was not directly measured in this study.

### 3.3. Depressive symptoms

Depressive symptoms were assessed using the PHQ-9, a widely validated measure of depressive symptom severity. Standard severity categories were applied: minimal (0–4), mild (5–9), moderate (10–14), moderately severe (15–19), and severe (20–27). Arabic versions of the PHQ-9 have demonstrated acceptable psychometric properties in regional populations.

### 3.4. Anxiety symptoms

Anxiety symptoms were measured using the GAD-7 scale. Severity categories followed standard thresholds: minimal (0–4), mild (5–9), moderate (10–14), and severe (15–21). Arabic versions of the GAD-7 have shown good reliability and validity in Middle Eastern settings.

### 3.5. Statistical analysis

Data were analyzed using International Business Machines Statistical Package for the Social Sciences Statistics (version 28). Descriptive statistics were calculated for all variables, including means and standard deviations (mean ± SD) for continuous scale scores and frequencies and percentages for categorical variables.

Associations between perceived social support (OSSS-3) and mental health outcomes (PC-PTSD-5, PHQ-9, and GAD-7) were examined using correlation analyses. Prior to analysis, assumptions for parametric testing were assessed through visual inspection and normality testing. Where assumptions were met, Pearson correlation coefficients were used; otherwise, Spearman rank correlations were applied. Statistical significance was set at *P* < .05 (two-tailed). Analyses were unadjusted; therefore, findings represent bivariate associations.

### 3.6. Ethical considerations

Ethical approval for the study was obtained from the Planning and Research Committee of the State Ministry of Health, Red Sea State, Port Sudan, Sudan. The study adhered to ethical principles for research involving conflict-affected populations, including voluntary participation, informed consent, confidentiality, and the right to withdraw at any time without consequences. Informed consent was obtained in Arabic, ensuring participants’ understanding of the study purpose and procedures.

## 4. Results

Out of the 207 IDPs residing in shelters in Port Sudan who participated in the study, the sample was composed of individuals aged 18 years and older. Participants were recruited using convenience sampling, meeting the eligibility criteria of age and residency in the shelters at the time of data collection. There were no reported exclusions due to nonparticipation or missing data on primary variables of interest, ensuring complete data availability for analysis.

### 4.1. Demographic and socioeconomic characteristics

The demographic and socioeconomic characteristics of participants reveal a predominantly adult, female, and low-income sample with significant challenges related to employment and economic stability. Most participants were adults aged 26 to 64 years (63.9%, n = 133), with young adults aged 18 to 25 years making up 33.2% (n = 69), and a smaller group of seniors aged 65 years and above comprising 2.9% (n = 6). The sample was predominantly female, with women representing 90.4% (n = 188) and men only 9.1% (n = 19). Nearly all participants were Sudanese nationals (98.6%, n = 205), with a small minority of foreign nationals (0.5%, n = 1). Employment and income data indicate that 84.1% (n = 175) of participants were unemployed, and 78.4% (n = 163) fell into the low-income category, while only 21.6% (n = 44) were classified as having average income. These findings underscore the socioeconomic vulnerabilities within the IDP population in Port Sudan (Table 1).

**Table 1:** Demographic and socioeconomic profile of study population.

Variable	Category	Frequency n (%)
Age group	Young adults (18–25 yr)	69 (33.2%)
	Adults (26–64 yr)	133 (63.9%)
	Seniors (65 yr and above)	6 (2.9%)
Gender	Female	188 (90.4%)
	Male	19 (9.1%)
Nationality	Non-Sudanese	1 (0.5%)
	Sudanese	205 (98.6%)
Marital status	Divorced	12 (5.8%)
	Married	122 (58.7%)
	Single	61 (29.3%)
	Widow	11 (5.3%)
Educational level	College/University	78 (37.5%)
	No formal education	11 (5.3%)
	Primary school	30 (14.4%)
	Secondary school	73 (35.1%)
	Post graduate	16 (7.7%)
Employment status	Unemployed	175 (84.1%)
	Employed	32 (15.4%)
Socio-economic status	Average income	44 (21.2%)
	Low income	163 (78.4%)

### 4.2. Descriptive statistics of mental health and social support measures

Overall levels of perceived social support and mental health symptoms were examined using mean scores and standard deviations. The mean Oslo Social Support Scale (OSSS-3) score was 7.69 ± 2.51, indicating generally low to moderate perceived social support. The mean PHQ-9 score was 9.86 ± 6.08, reflecting a moderate level of depressive symptoms. The mean GAD-7 score was 8.46 ± 5.53, corresponding to mild-to-moderate anxiety symptoms. The mean Primary Care PTSD Screen for DSM-5 (PC-PTSD-5) score was 1.98 ± 1.40, indicating a substantial burden of post-traumatic stress symptoms within the sample.

### 4.3. Distribution of social support across mental health severity categories

**Figure 1.**
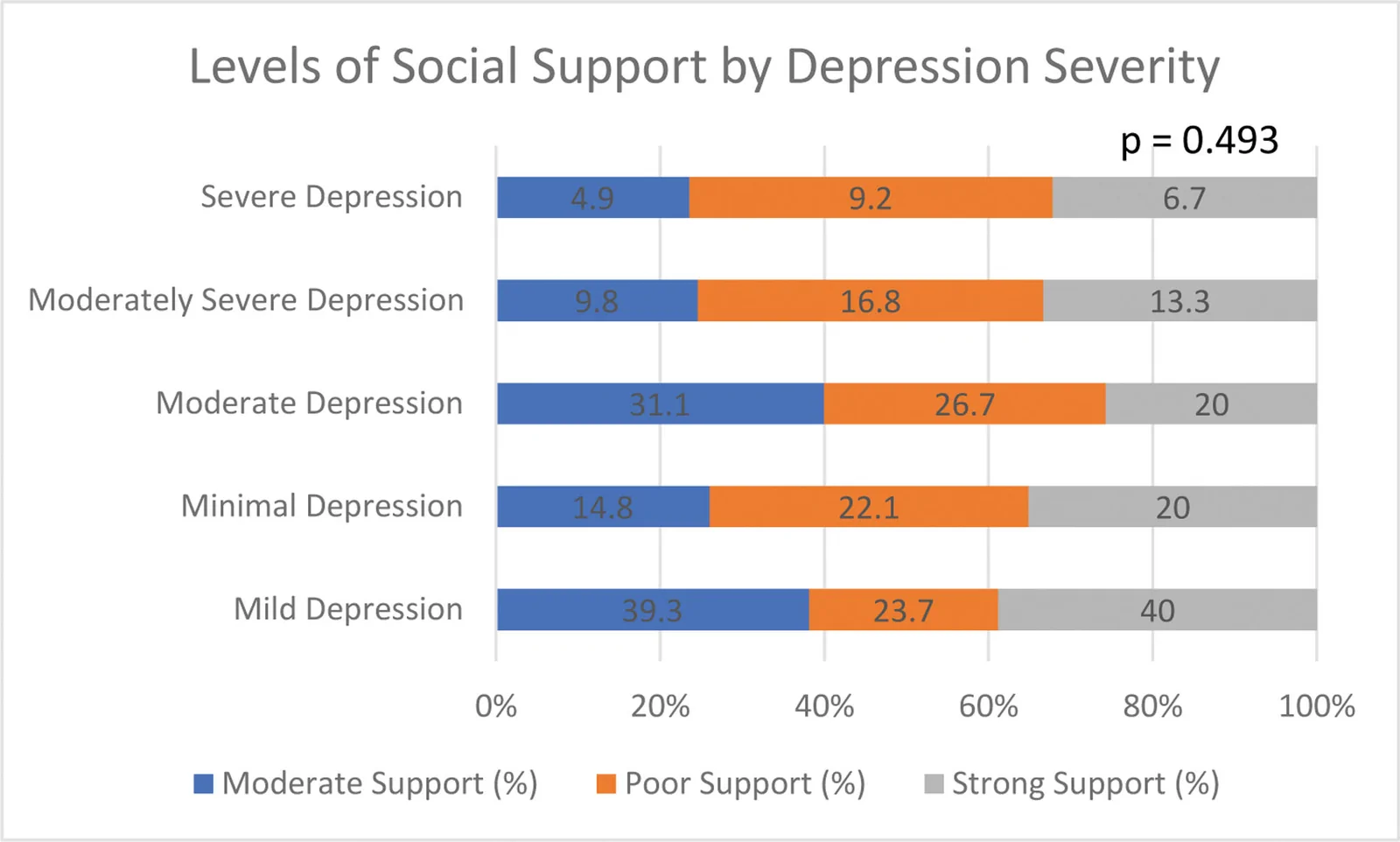
Social support levels across depression severity categories.

The distribution of social support levels across depression severity categories is shown in Figure 1. Among participants with mild depression (30.5%, n = 61), 40% reported strong support, 23.7% reported poor support, and 39.3% reported moderate support. In contrast, participants with severe depression (8%, n = 16) showed lower levels of strong support (6.7%) and moderate support (4.9%), with 9.2% reporting poor support.

**Figure 2.**
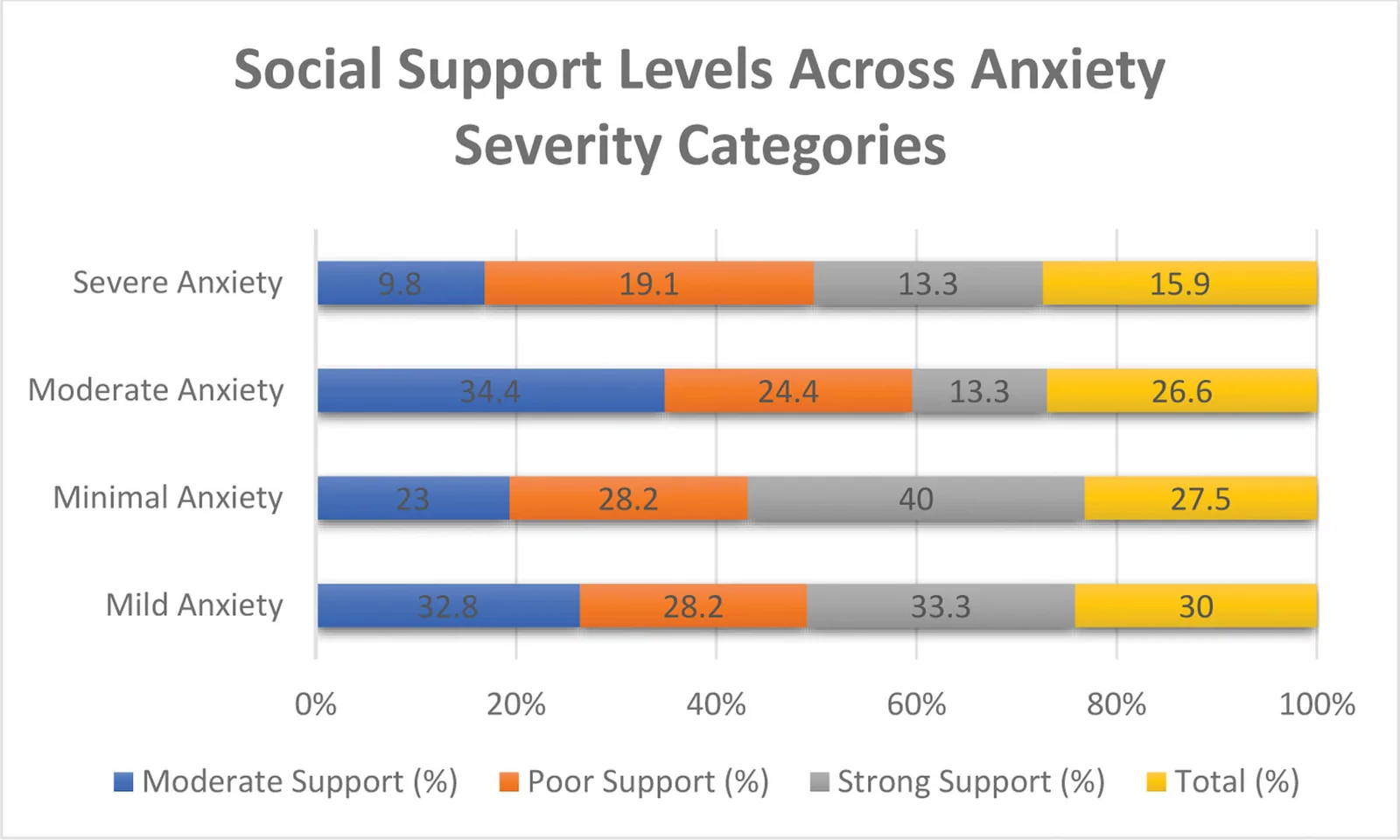
Social support levels across anxiety severity categories.

Social support levels were also examined across anxiety severity categories, as illustrated in Figure 2. Participants with mild anxiety (31%, n = 62) reported strong support in 33.3% of cases, moderate support in 32.8%, and poor support in 28.2%. Those with severe anxiety (16.5%, n = 33) reported strong support in 13.3% of cases, moderate support in 9.8%, and poor support in 19.1%.

**Figure 3.**
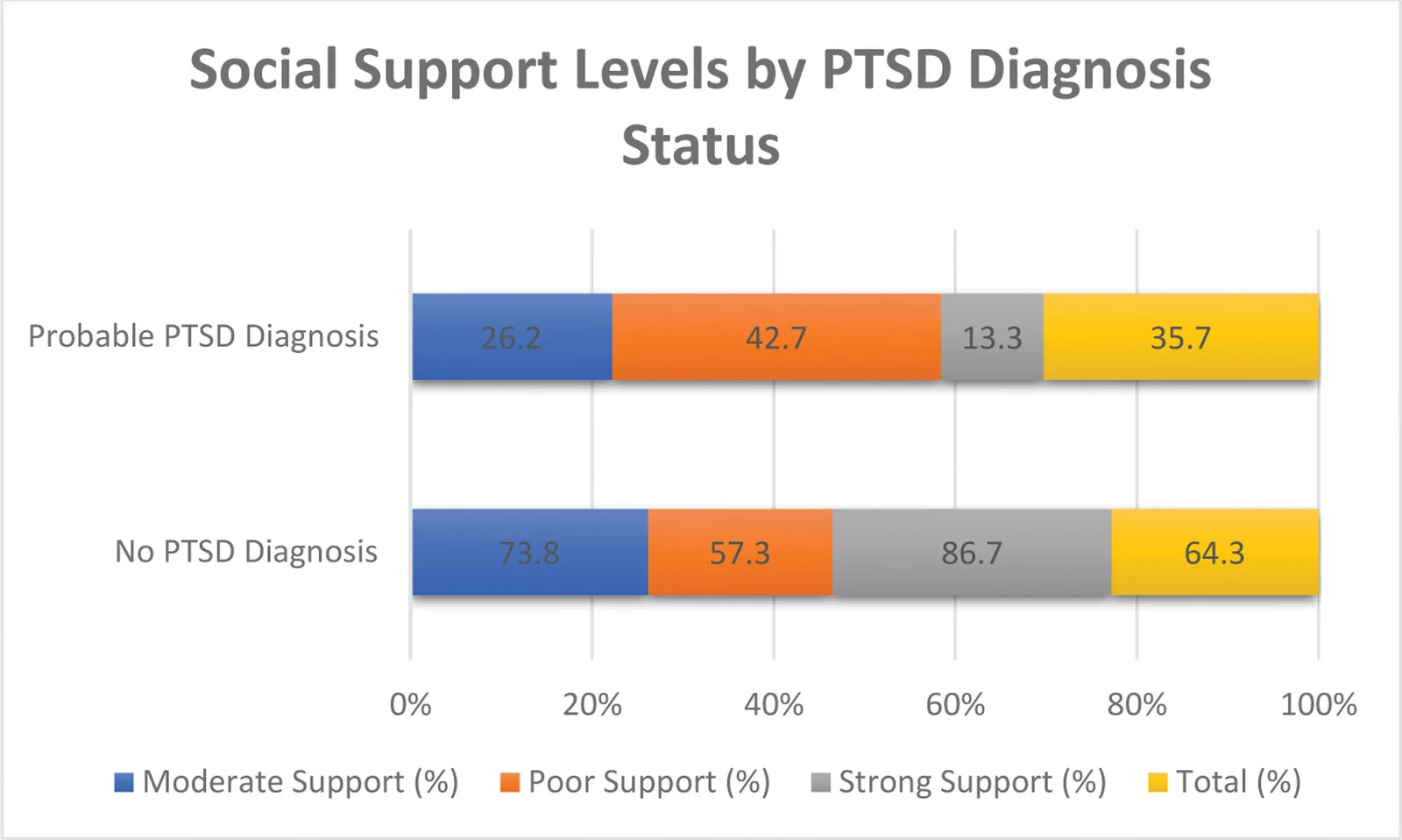
Social support levels among individuals with and without PTSD diagnosis. PTSD = post-traumatic stress disorder.

The analysis of social support levels among individuals with and without a probable PTSD diagnosis is shown in Figure 3. Participants with probable PTSD (37%, n = 74) were more likely to report poor social support (42.7%) and less likely to report strong support (13.3%) compared to participants without PTSD (63%, n = 126), who reported poor support in 57.3% of cases and strong support in 86.7%.

### 4.4. Correlations between social support and mental health outcomes

**Figure 4.**
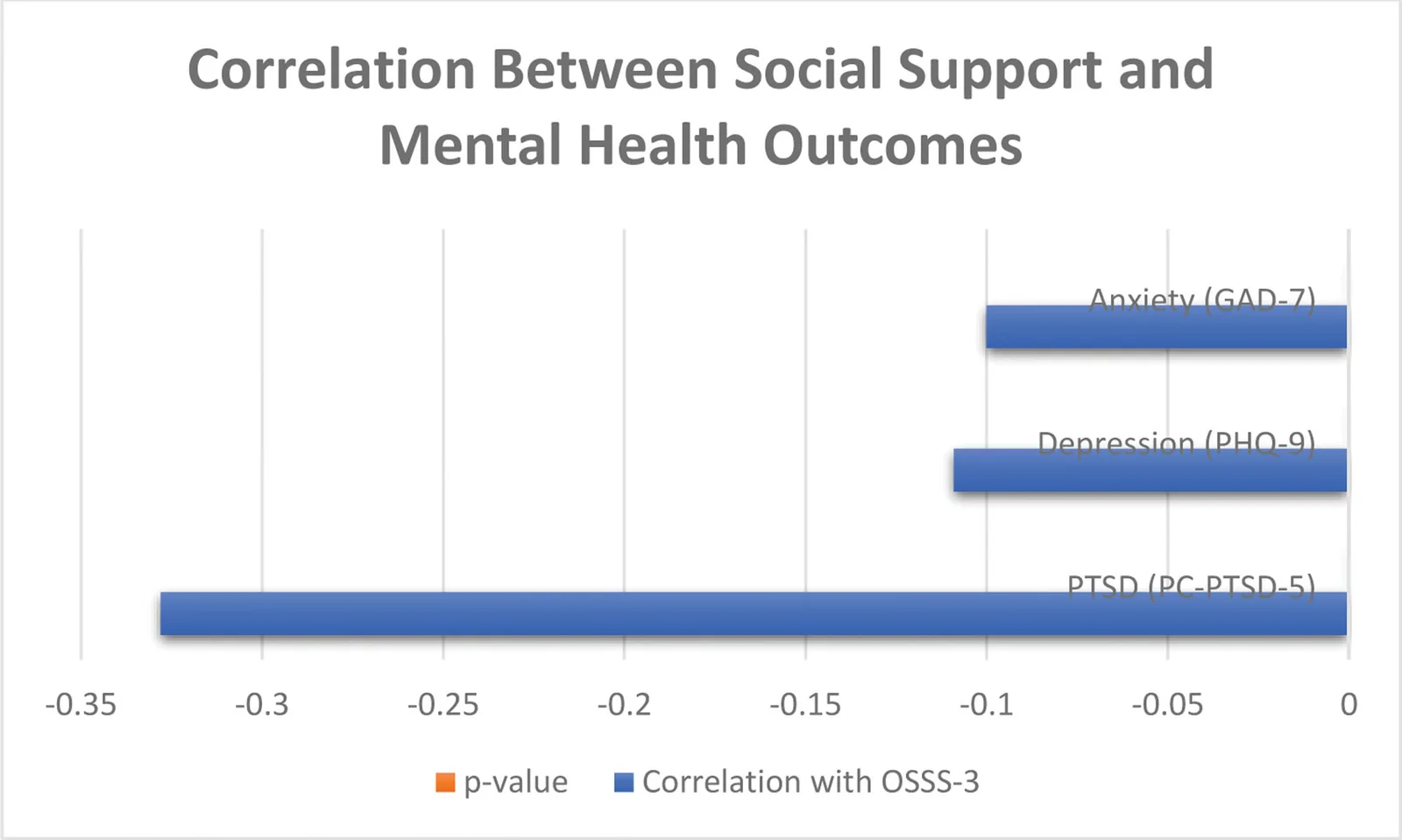
Impact of social support on mental health outcomes: correlation analysis.

**Figure 5.**
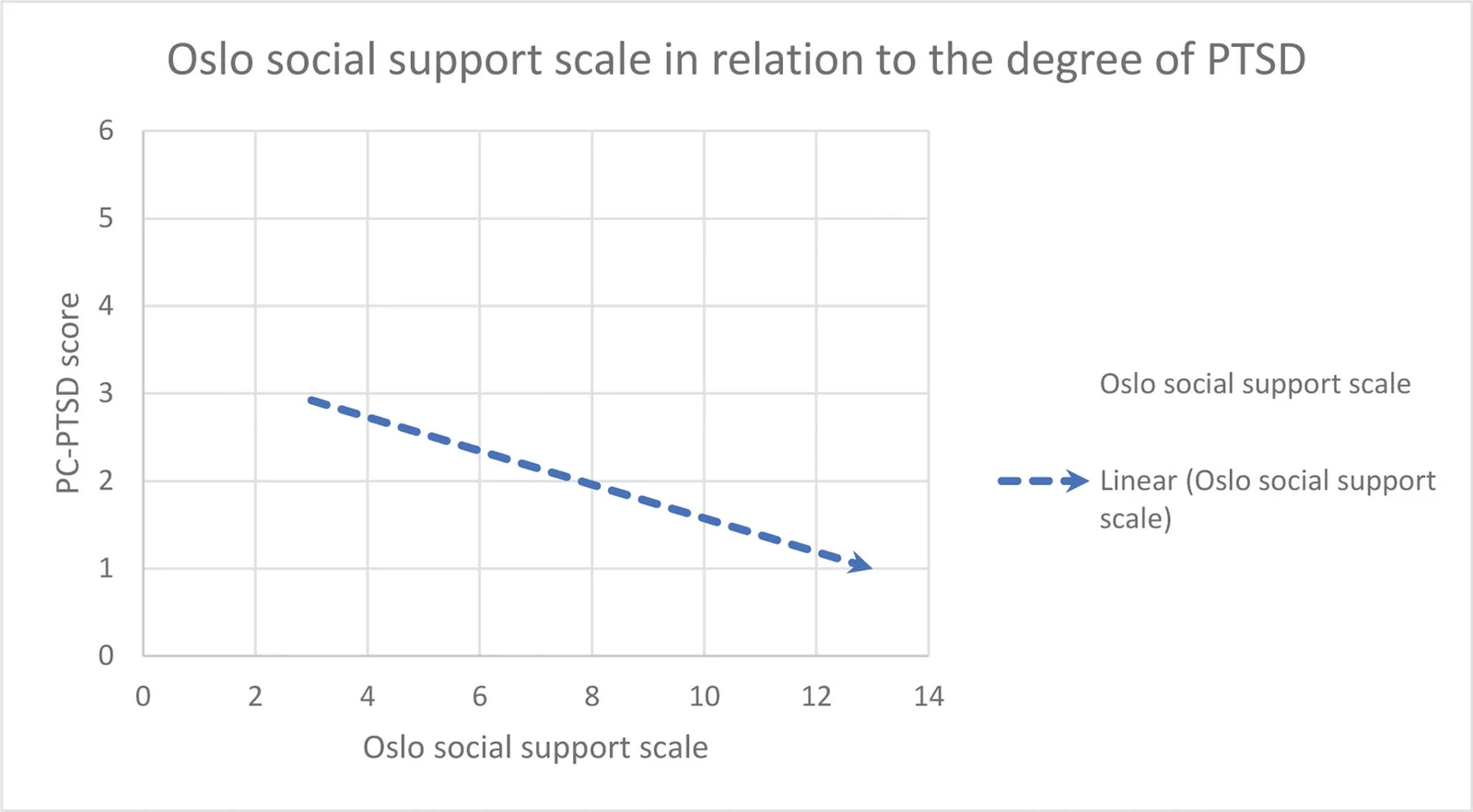
Scatter plot of social support and PTSD symptom scores. PTSD = post-traumatic stress disorder.

Correlations between perceived social support (OSSS-3) and mental health outcomes (PTSD, depression, and anxiety) were examined. Figure 4 presents the correlation between social support and post-traumatic stress symptoms, while Figure 5 displays the scatter plot with individual participant data points and fitted trendline.

A moderate, statistically significant negative correlation was observed between OSSS-3 scores and PC-PTSD-5 scores (*r* = −0.328, *P* < .01), indicating that higher perceived social support was associated with fewer post-traumatic stress symptoms. Participants without probable PTSD were more likely to report moderate (73.8%) or strong social support (86.7%), whereas those with probable PTSD were more likely to report poor support (42.7%) (Figs. 4 and 5).

In contrast, weak and non-significant negative correlations were observed between perceived social support and depression (PHQ-9; *r* = −0.109, *P* = .118) and anxiety (GAD-7; *r* = −0.100, *P* = .152), indicating limited association with these outcomes.

All correlation analyses were conducted without adjustment for demographic variables such as age or gender.

### 4.5. Association between social support and receipt of psychological health support treatment

**Figure 6.**
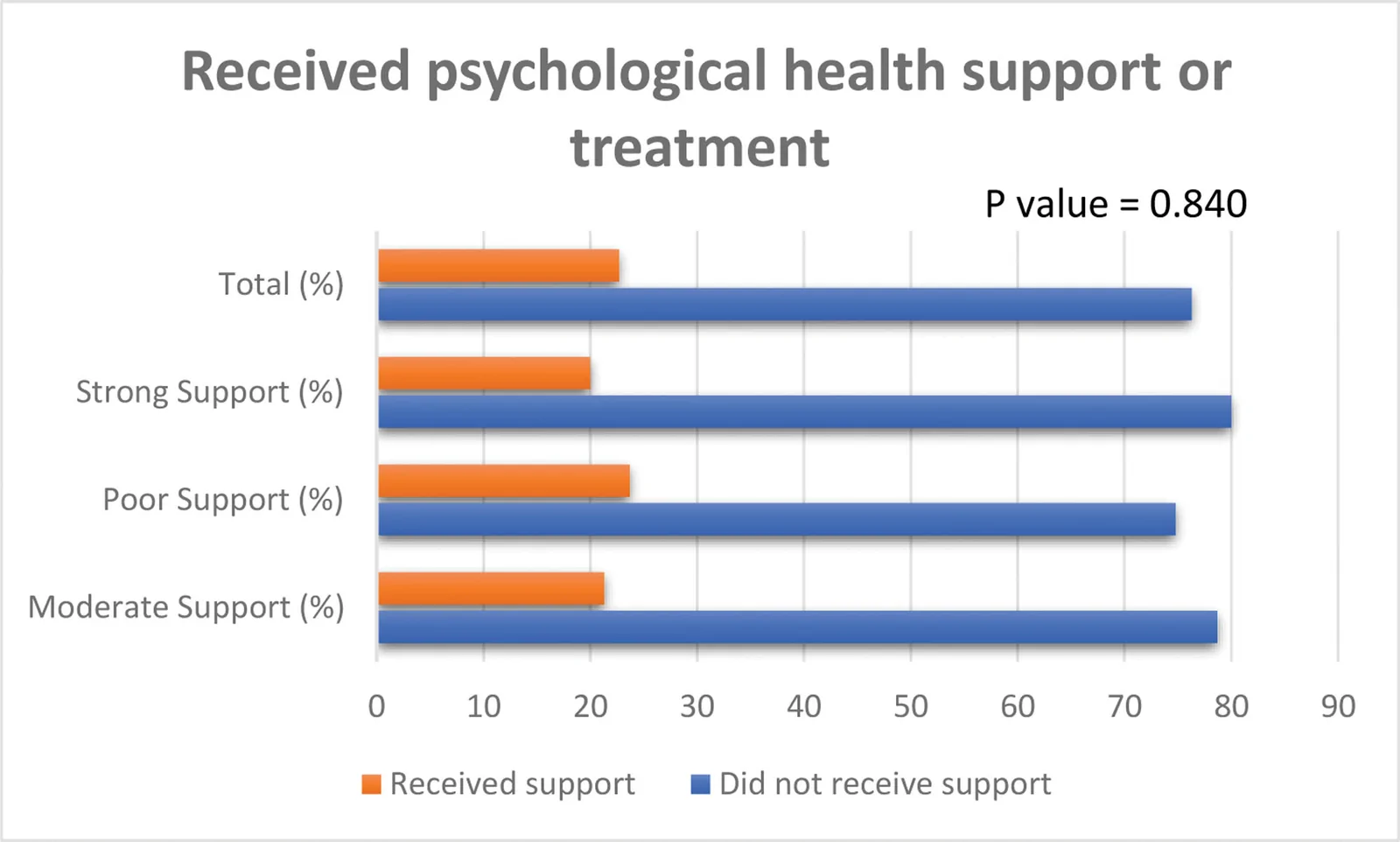
Distribution of social support levels among displaced individuals receiving psychological health support.

The association between perceived social support levels and receipt of psychological health support is presented in Figure 6. Among participants with strong social support, 80.0% did not receive psychological health support, while 20.0% did. Similarly, 74.8% of participants with poor social support and 78.7% of those with moderate support did not receive psychological health support.

There was no statistically significant association between perceived social support level and receipt of psychological health support (*P* = .840), indicating that social support was not associated with service utilization in this setting.

## 5. Discussion

This study provides context-specific insight into how social support relates differently to post-traumatic stress symptoms, depression, anxiety, and service utilization among internally displaced adults living in shelters in Port Sudan. Rather than suggesting uniform mental health vulnerability among displaced populations, the findings highlight a differentiated pattern: social support appears particularly salient for post-traumatic stress symptoms, while depressive and anxiety symptoms, as well as engagement with psychological services, are more strongly shaped by structural, socioeconomic, and systemic factors. This distinction is critical for designing humanitarian MHPSS responses that are both effective and non-pathologizing.^[16]^

A moderate and statistically significant negative association was observed between perceived social support and post-traumatic stress symptoms (*r* = −0.328, *P* < .01). Participants screening positive for probable PTSD (37%, n = 74) were substantially more likely to report poor social support (42.7%) and less likely to report strong support (13.3%) than those screening negative (63%, n = 126), among whom 86.7% reported strong support. This pattern suggests that lower perceived social support is associated with greater post-traumatic stress symptom burden among internally displaced adults in this setting. These findings are consistent with the stress-buffering hypothesis, which posits that social relationships can attenuate the psychological impact of stressors by enhancing emotional regulation, adaptive cognitive appraisal, and coping capacity.^[17]^ Within humanitarian frameworks, these results also align with Inter-Agency Standing Committee guidance, which identifies social and community supports as foundational components of MHPSS responses in emergency settings.^[16]^

In the Sudanese displacement context, the observed association between social support and post-traumatic stress symptoms may reflect mechanisms that are particularly salient to threat-related psychopathology. Post-traumatic stress symptoms are closely linked to disruptions in safety regulation, persistent hyperarousal, and intrusive threat processing.^[13]^ Interpersonal support may function as a “social safety signal,” facilitating emotional co-regulation, shared meaning-making, and restoration of predictability in environments characterized by insecurity, loss, and uncertainty.^[11]^ In overcrowded shelter settings where exposure to stressors is often ongoing rather than confined to past events the presence of trusted social ties may reduce both physiological and psychological stress reactivity by reinforcing a sense of protection, belonging, and collective coping. Evidence from other conflict-affected and displaced populations in Africa similarly indicates that social connectedness is more consistently associated with post-traumatic stress symptoms than with other common mental health outcomes, supporting the plausibility of the observed pattern.^[7]^

In contrast, associations between perceived social support and depression (*r* = −0.109) and anxiety (*r* = −0.100) were weak and not statistically significant. This divergence likely reflects differences in underlying pathways through which social and contextual factors influence mental health outcomes in displacement settings. In situations of protracted displacement, depressive and anxiety symptoms are frequently sustained by chronic stressors, including unemployment, income insecurity, prolonged uncertainty, caregiving burden, and diminished personal agency, which may persist even when supportive interpersonal relationships are present.^[6]^ In the present sample, socioeconomic adversity was pronounced, with 84.1% of participants unemployed and 78.4% reporting low income. Such conditions are well documented as key drivers of depression and anxiety in forced displacement settings and may overwhelm the protective effects of social support alone.^[18]^

Nevertheless, descriptive gradients suggest that social support may still be relevant at the level of symptom severity. Only 6.7% of participants with severe depression (8%, n = 16) and 13.3% of those with severe anxiety (16.5%, n = 33) reported strong social support, compared with 40% and 33.3%, respectively, among those with mild symptom levels. This pattern suggests attenuation rather than absence of effect, which is consistent with evidence from other African displacement contexts indicating that structural deprivation and chronic adversity may moderate the relationship between psychosocial resources and mental health outcomes.^[15]^

The pronounced gender imbalance in the sample (90.4% female) warrants interpretation beyond methodological considerations alone. Displaced women in Sudan frequently experience intersecting vulnerabilities related to caregiving responsibilities, restricted access to livelihoods, heightened exposure to gender-based violence, and limited decision-making power.^[19]^ These overlapping social and economic constraints may intensify psychological distress while simultaneously constraining opportunities for recovery. Gender, socioeconomic position, and displacement status therefore interact to shape mental health outcomes, reinforcing the importance of intersectional and gender-sensitive MHPSS programming rather than narrowly individualized or symptom-focused approaches.^[6]^

A particularly important finding is that perceived social support was not associated with utilization of psychological health services (*P* = .840). Across all levels of social support, the majority of participants did not receive psychological care (80% among those with strong support, 74.8% among those with poor support, and 78.7% among those with moderate support). This pattern suggests that service utilization is constrained less by interpersonal resources and more by systemic and sociocultural barriers. Beyond physical access, such barriers likely include limited service availability, insecurity and transportation difficulties, financial constraints, low awareness of services, concerns regarding confidentiality, stigma, and limited trust in formal health systems.^[20]^ In Sudan, conflict-related damage to health infrastructure and shortages of trained personnel have further undermined service continuity, particularly for mental health care.^[9]^ In this context, social networks, while potentially protective at the psychosocial level, are insufficient to overcome layered structural barriers without deliberate system-level interventions.

Importantly, the relevance of social support observed in this study should be interpreted within the cultural and social fabric of Sudanese communities, where collective solidarity, extended kinship relations, neighborly assistance, and religiously grounded mutual aid are longstanding norms rather than externally introduced constructs.^[21,22]^ During periods of political instability and economic hardship, Sudanese communities have repeatedly relied on informal support structures and community self-organization to meet basic needs and protect social cohesion, including in the current conflict through locally organized mutual-aid initiatives such as emergency response rooms and community-led service provision.^[23,24]^ In displacement settings, these culturally embedded forms of social support may retain particular psychological salience even as formal institutions weaken, providing continuity, shared identity, and collective meaning-making that can support coping and reduce threat-related distress.^[21]^ Conceptualising social support as a culturally rooted resource, rather than solely a programmatic “intervention,” aligns with humanitarian principles that emphasize dignity, local capacities, and community resilience, and is consistent with guidance that places community and family supports at the foundation of emergency MHPSS responses.^[16]^

Taken together, these findings support a layered, community-based approach to MHPSS in displacement settings. Strengthening social connectedness within shelters through peer support initiatives, women’s groups, youth activities, and community-led programmes may be particularly relevant for post-traumatic stress symptoms, while depressive and anxiety symptoms require integrated responses that also address chronic material deprivation and structural stressors. The absence of an association between social support and service utilization further underscores the need for outreach-oriented, culturally acceptable, and trust-building service delivery models, including task-sharing and stepped-care approaches aligned with the WHO Mental Health Gap Action Programme Humanitarian Intervention Guide.^[10]^ Such approaches are consistent with international humanitarian guidance and offer a pragmatic strategy for reducing the mental health treatment gap without pathologizing displaced populations.

### 5.1. Key implications for practice

- Humanitarian MHPSS programmes in displacement shelters should prioritize strengthening social connectedness and peer, family, and community support structures, as higher perceived social support was associated with fewer post-traumatic stress symptoms among internally displaced adults.
- Because perceived social support was not associated with utilization of psychological health services, humanitarian actors should address structural and sociocultural barriers to care – such as service availability, safe referral pathways, stigma, trust, and awareness – alongside community-based psychosocial supports.
- In low-resource conflict settings, integrating culturally grounded, community-based psychosocial interventions with scalable, non-specialist and task-shared care models (e.g., Mental Health Gap Action Programme-aligned approaches) may promote wellbeing, coping, and resilience without pathologizing displaced populations.

### 5.2. Limitation

This study is subject to several limitations. The cross-sectional design restricts causal inference, and the observed relationships between social support and mental health outcomes should be interpreted as associative rather than directional. Post-traumatic stress symptoms were assessed using the PC-PTSD-5, while trauma exposure itself was not directly measured; accordingly, findings relate to screened post-traumatic stress symptoms or probable PTSD rather than confirmed exposure or clinical diagnosis. The use of convenience sampling limits generalisability, and the marked gender imbalance (90.4% female) constrains applicability to displaced men. Furthermore, the sample reflects conditions within selected shelters at a specific point in time rather than the broader internally displaced population. Finally, although utilization of psychological health services was examined, service availability, quality, and sociocultural barriers were not assessed, and reliance on self-reported measures may introduce reporting bias despite the use of validated instruments.

## 6. Conclusion

This study provides evidence from Port Sudan that perceived social support is differentially associated with mental health outcomes among internally displaced adults living in shelters. Higher social support was associated with fewer post-traumatic stress symptoms, whereas associations with depression, anxiety, and utilization of psychological health services were weak or non-significant. These findings highlight heterogeneity in displacement-related distress and caution against conceptualizing IDPs as a uniformly traumatized population.

From a policy and practice perspective, the findings underscore the relevance of community-based, resilience-oriented MHPSS approaches that strengthen social connectedness and collective coping. At the same time, the absence of an association between social support and service utilization points to persistent structural and sociocultural barriers that require health-system and humanitarian responses beyond interpersonal support alone. Integrating culturally grounded community initiatives with accessible, task-shared care models embedded within humanitarian and primary health platforms may offer a feasible pathway to improving wellbeing in low-resource, conflict-affected settings.

Future research should prioritize longitudinal and mixed-methods designs to clarify temporal relationships, incorporate direct measures of trauma exposure and service availability, and explore how culturally embedded forms of social support interact with structural constraints over time. Such work is essential to inform scalable interventions that build on local capacities and promote coping, recovery, and wellbeing among conflict-affected populations without reinforcing pathologizing narratives.

## Acknowledgments

The author expresses his deepest gratitude to the internally displaced persons who participated in this study, sharing their experiences and insights despite the immense challenges they face. Special thanks are extended to the data collection team for their dedication and professionalism in ensuring the accuracy and integrity of the research process. Acknowledgments is also given to the local authorities in Port Sudan for granting ethical approval and facilitating the study’s implementation.

## Author contributions

**Conceptualization:** Mohammed Salah Alfahal.

**Data curation:** Mohammed Salah Alfahal.

**Formal analysis:** Mohammed Salah Alfahal.

**Methodology:** Mohammed Salah Alfahal.

**Project administration:** Mohammed Salah Alfahal.

**Resources:** Mohammed Salah Alfahal.

**Software:** Mohammed Salah Alfahal.

**Supervision:** Mohammed Salah Alfahal.

**Validation:** Mohammed Salah Alfahal.

**Visualization:** Mohammed Salah Alfahal.

**Writing – original draft:** Mohammed Salah Alfahal.

**Writing – review & editing:** Mohammed Salah Alfahal.
